# Activation of Membrane Estrogen Receptors Attenuates NOP-Mediated Tactile Antihypersensitivity in a Rodent Model of Neuropathic Pain

**DOI:** 10.3390/brainsci9060147

**Published:** 2019-06-21

**Authors:** Danyeal M. Wright, Keri M. Small, Subodh Nag, Sukhbir S. Mokha

**Affiliations:** Department of Biochemistry, Cancel Biology, Neuroscience and Pharmacology, Meharry Medical College, Nashville, TN 37208, USA; dheckard@mmc.edu (D.M.W.); kerimcleansmall@gmail.com (K.M.S.); smokha@mmc.edu (S.S.M.)

**Keywords:** nociceptin/orphanin FQ receptor, neuropathic pain, spinal cord, spared nerve injury, analgesia

## Abstract

Women manifest a higher prevalence of several chronic pain disorders compared to men. We demonstrated earlier that estrogen rapidly attenuates nociceptin/orphanin FQ (N/OFQ) peptide receptor (NOP)-mediated thermal antinociception through the activation of membrane estrogen receptors (mERs). However, the effect of mER activation on NOP-mediated attenuation of tactile hypersensitivity in a neuropathic model of pain and the underlying mechanisms remain unknown. Following spared nerve injury (SNI), male and ovariectomized (OVX) female rats were intrathecally (i.t.) injected with a selective mER agonist and nociceptin/orphanin FQ (N/OFQ), the endogenous ligand for NOP, and their effects on paw withdrawal thresholds (PWTs) were tested. In addition, spinal cord tissue was used to measure changes in phosphorylated extracellular signal regulated kinase (ERK), protein kinase A (PKA), protein kinase C (PKC), and protein kinase B (Akt) levels. SNI significantly reduced PWTs in males and OVX females, indicating tactile hypersensitivity. N/OFQ restored PWTs, indicating an antihypersensitive effect. Selective mER activation attenuated the effect of N/OFQ in an antagonist-reversible manner. SNI led to a robust increase in the phosphorylation of ERK, PKA, PKC, and Akt. However, mER activation did not further affect it. Thus, we conclude that activation of mERs rapidly abolishes NOP-mediated tactile antihypersensitivity following SNI via an ERK-, PKA-, PKC-, and Akt-independent mechanism.

## 1. Introduction

Opiates acting at the µ-opioid receptor have been the most effective and most commonly used analgesics to treat severe pain conditions, e.g., neuropathic and inflammation-induced pain. However, they are associated with many adverse side effects, including tolerance, dependence, and constipation [1]. The nociceptin/orphanin FQ (N/OFQ) peptide receptor (NOP), a G protein-coupled receptor (GPCR), is a relatively newly discovered member of the opioid receptor family [2,3]. Preclinical studies have shown that activation of the NOP receptor is associated with fewer deleterious side effects than that of other opioid receptors [4,5,6]. The NOP, as well as its endogenous ligand N/OFQ, is expressed in the dorsal horn of the spinal cord and other pain processing areas of the brain [2,3,7]. Upon activation, NOP couples to inhibitory G proteins (G_i/o_) to initiate a signaling cascade that facilitates G protein-coupled inwardly rectifying potassium (GIRK) channel function, causing neuronal hyperpolarization and ultimately leading to decreased nociceptive signaling [8]. Sex-related differences in pain have been reported, with women having a higher prevalence of several pain disorders, e.g., fibromyalgia, migraine headaches, and temporomandibular joint disorder (TMJD), compared to men [9,10,11,12]. Preclinical studies, including our own, have also revealed estrogen-induced reduction of GPCR-mediated analgesia in females [13,14,15,16,17]. We recently reported that NOP-mediated thermal antinociception in an acute pain model was quickly diminished following the activation of membrane estrogen receptors (mERs) GPR30, Gq-mER, and ERα, but not ERβ [18]. However, selective contribution of each mER to the attenuation of NOP-mediated tactile antihypersensitivity in a neuropathic pain model is not known. Therefore, this study investigated the effect of spinal mER activation on NOP-mediated tactile antihypersensitivity following spared nerve injury (SNI). Since mERs have been shown to activate several kinases [18,19] that may modulate GIRK function, we also measured spinal levels of activated PKA, PKC, Akt, and ERKI/II in response to spinal mER activation. 

## 2. Materials and Methods

### 2.1. Animals

Adult Sprague Dawley male and ovariectomized (OVX) female rats (250–274 g) were obtained from Envigo (Envigo, Indianapolis, IN, USA). Animals were housed in the Meharry Medical College Animal Care Facility (ACF), which is qualified by the American Association for the Accreditation of Laboratory Animal Care (AAALAC), under a 12-h light/dark cycle (lights on 7:00–19:00). Food and water were available ad libitum. The experimental protocols were accepted by the Institutional Animal Care and Use Committee of Meharry Medical College and abided by the conventional guidelines of the National Research Council Guide for the Care and Use of Laboratory Animals and the International Association for the Study of Pain (IASP). All efforts were made to minimize stress to animals and the number of animals used. A total of 655 animals were used to complete the behavior and molecular experiments in this study. 

### 2.2. Implantation of Cannulae 

OVX female animals were given a 2-week recovery period prior to surgery. As described [20], animals were anesthetized with an intraperitoneal (i.p.) injection of ketamine (72 mg/kg) and xylazine (4 mg/kg). Using aseptic surgical procedures, the head and left hind leg were shaved, and the skin was disinfected with alternating scrubs of ethanol (70%) and betadine (10%). Their heads were then secured in a stereotaxic frame (David Kopf Instruments, Tujunga, CA, USA). An incision was made above the head/neck area, and the atlanto-occipital membrane was removed to expose the dura. A stretched, sterile PE-10 cannula (Intramedic, Clay Adams, Sparks, MD, USA; dead space volume 10 μL) was implanted into the subarachnoid space through a small opening in the dura. The cannula was pushed to a length of 9.0 cm to reach the lumbosacral enlargement. The cannula was secured by dental cement, and the wound was closed with suture clips. The position of the cannula was confirmed at the end of the experiment by administering 10 μL of 2% lidocaine (i.t.), which temporarily paralyzed the animals’ hind limbs, and through a visual examination of Chicago sky blue dye (Sigma, St. Louis, MO, USA) spread. In this study, no animals were excluded due to incorrect cannula positioning. Animals used for immunoblotting were not administered lidocaine or blue dye to minimize sample contamination. Instead, cannula placement was confirmed by the observation of a drug effect and visual inspection during dissection for sample collection. 

### 2.3. Spared Nerve Injury

For the modeling of neuropathic pain, the spared nerve injury (SNI) of the sciatic nerve has been previously described [21]. Following intrathecal (i.t.) cannulation, a small longitudinal incision was made proximal to the left knee, and the skin and underlying muscle were retracted by blunt dissection until the sciatic nerve was exposed at the trifurcation into the sural, tibial, and common peroneal nerves. The tibial and common peroneal nerves were tightly ligated and severed, leaving the sural nerve intact. The overlying muscle was then sutured, and the overlying skin was secured with suture clips. Animals in the sham group had their sciatic nerve exposed and muscle/skin sutured, as in the SNI procedure, but received no further manipulation. Animals were kept warm on a heating blanket until they regained consciousness and returned to ACF. They were allowed to recover for 7 days before nociceptive testing. Animals were monitored daily for any sign of neurological deficits and overall health. Animals displaying any neurological impairment were euthanized. Twenty-four animals were excluded due to neurological impairments.

### 2.4. Paw Withdrawal Assay 

Tactile hypersensitivity was assessed on day 7 following surgery using an automated dynamic plantar aesthesiometer (Model 37400; Ugo Basile, Comerio, Italy). Animals were placed in a plastic cage with a wire mesh floor and were allowed to acclimate for at least 30 min before behavior testing. The machine applied a metal filament (0.5 mm diameter) to the lateral plantar surface (the region innervated by the “spared” sural nerve) of the left hind paw and applied an increasing force until the paw was withdrawn or the preset cutoff was reached (50 g). The force applied was originally below the detection threshold, and then increased at a rate of 2.5 g/s. The force required to provoke withdrawal was recorded automatically. Three baseline mechanical thresholds were recorded at 2-min intervals, and the testing continued for 20 min post-drug injection. 

### 2.5. Drugs

Each drug was injected intrathecally (5-s time span) via the implanted cannula with a 50-μL Hamilton microsyringe in a volume of 10 μL at time “0”, unless stated otherwise. The dose (10 nmol) of N/OFQ, the endogenous ligand for NOP, was selected based on our previously reported dose-response curves, which produced a robust antinociceptive effect in the tail flick assay [13]. E_2_BSA (β-estradiol 6-(*O*-carboxymethyl) oxime/bovine serum albumin (BSA)), a membrane impermeant analog of estradiol, was administered to target all membrane estrogen receptors. The E_2_BSA dose (0.5 mM) was chosen based on our previous study [18,20] and other [22,23] studies. Doses of propylpyrazoletriol (PPT), an ERα-selective agonist, and diarylpropionitrile (DPN), an ERβ-selective agonist (100 nM), were selected based on previous reports [18,24]. G-1 is a selective agonist for the GPR30 receptor: The 0.25-nM dose was based on the binding affinity of G-1 to GPR30 [25]. STX (10 nM) is a Gq-mER selective agonist with ~20× higher affinity than E_2_ [26,27]. G-15 (1 μM), a GPR30 antagonist, was injected 5 min prior to G-1. N/OFQ, G-1, G15, PPT, and DPN were acquired from Tocris (Ellisville, MO, USA), whereas E_2_BSA was acquired from Sigma-Aldrich (St. Louis, MO, USA). Dr. Martin Kelly at Oregon Health Sciences University kindly provided STX. Drugs were dissolved in phosphate-buffered saline (PBS) (E_2_BSA), double-distilled boiled water (N/OFQ), <1% ethanol (G-15, PPT, DPN), or <10% dimethyl sulfoxide (DMSO) (G1 and STX). Prior to intrathecal administration, E_2_BSA was centrifuged at 13,000× *g* for 30 min in a 0.5-mL Microcon Cartridge (Millipore, Temecula, CA, USA) to remove any unbound E_2_, as previously described by Stevis et al. in 1999 [28]. We successfully used the above-described ligands at exact doses in our previously published study [18]. Proper vehicles were used to control for the drug as well as volume effects, which were not significantly different from pre-drug baseline paw withdrawal latencies.

### 2.6. Immunoblotting 

Lumbosacral spinal cords of anesthetized (0.04 kg/mg Beuthanasia) SNI and sham rats were collected ~10 min following in vivo i.t. E_2_BSA, N/OFQ, or E_2_BSA + N/OFQ treatment. Drug effects on paw withdrawal thresholds (PWTs) were behaviorally confirmed at 3 time points in the paw withdrawal assay. Tissues were kept in 0.5 mL of RNAlater (Ambion, Austin, TX, USA) at −80 °C until further analysis. Tissue homogenates were prepared in 0.5 mL of radioimmunoprecipitation assay buffer (RIPA) lysis buffer (Santa Cruz Biotech, Dallas, TX, USA) containing tris-buffered saline (TBS), 1% Nonidet P-40, 0.5% sodium deoxycholate, 0.1% sodium dodecyl sulfate (SDS), and 0.004% sodium azide. Phenylmethylsulfonyl fluoride (PMSF), sodium orthovanadate, and protease inhibitor cocktail were added to RIPA (10 μL/mL) immediately before use. Total protein contents were evaluated using a Lowry [29] assay-based detergent-compatible (DC) reagent kit (Bio-Rad, Hercules, CA, USA). SDS-PAGE was run with the NuPAGE gel system (Life Technologies, Grand Island, NY, USA): Samples were processed per the manufacturer’s guidelines, heated at 65 °C for 10 min, and loaded onto the gel. Proteins were transferred onto PVDF membrane and processed for immunoblotting using selective primary antibodies against PKA, pPKA (Upstate, Lake Placid, NY, USA), PKC, pPKC (Pierce, Rockford, IL, USA), ERK I/II, pERK I/II (Cell Signaling Technology Inc., Danvers, MA, USA), Akt, pAkt (1:1000, Cell Signaling Technology, Danvers, MA, USA), and actin (1:1000, Sigma, St. Louis, MO, USA). All incubations were carried out in closed containers on Belly Dancer orbital shakers (Stovall, Greensboro, NC, USA). Blots were first blocked with 5% nonfat dairy milk in tris-buffered saline containing 0.05% Tween 20 (TBST; Santa Cruz) for 1 h and were then incubated with primary antibody for 12–48 h on a shaker at 4 °C. After washing, the blots were incubated for 1 h at room temperature with horseradish peroxidase (HRP)-conjugated secondary antibody (bovine antirabbit IgG-HRP, 1:7500, Sigma, St. Louis, MO, USA), washed, and developed using Super Signal West Dura Extended Duration^®^ (Thermo Scientific, Waltham, MA, USA) for 5 min. Immunopositive bands were imagined with a Gel Doc System (UVP, LLC, Upland, CA, USA), and images were stored for densitometry analysis using LabWorks 4.6 (UVP) software (Bio-Rad, Hercules, CA, USA). The data were normalized against actin and are presented as normalized phosphoprotein/total protein.

### 2.7. Data Analysis

Data were analyzed using SPSS (SPSS Inc., Chicago, IL, USA) and Prism (Graphpad Software, Inc., San Diego, CA, USA). Data were first checked for normal distribution using the Shapiro–Wilk normality test in Prism. The analysis indicated that the dataset, across all groups, was indeed normally distributed (minimum *W* = 0.778; passed normality test). All behavior measures were submitted to an ANOVA corrected for repeated measures with proper between-group (sex, drug) and within-group (time) factors and dependent variables (PWTs). The number of animals in each group was 3–6. The area under the curve (AUC) was calculated through the trapezoid method using Prism (Graphpad Software, Inc., San Diego, CA, USA) for time course plots to attain a single measure of the total drug response. The data acquired from western blotting studies and the AUC were analyzed by one-way ANOVA. A Bonferroni post hoc test was employed for intergroup comparisons where needed and only when ANOVA yielded a significant main effect. A *p*-value < 0.05 was considered significant. Data were plotted as mean ± S.E.M. using Prism (Graphpad Software, Inc., San Diego, CA, USA). 

## 3. Results

### 3.1. N/OFQ Reversesd Tactile Hypersensitivity following SNI, and E_2_-BSA Rapidly Attenuated the Effect of N/OFQ

**Figure 1:**
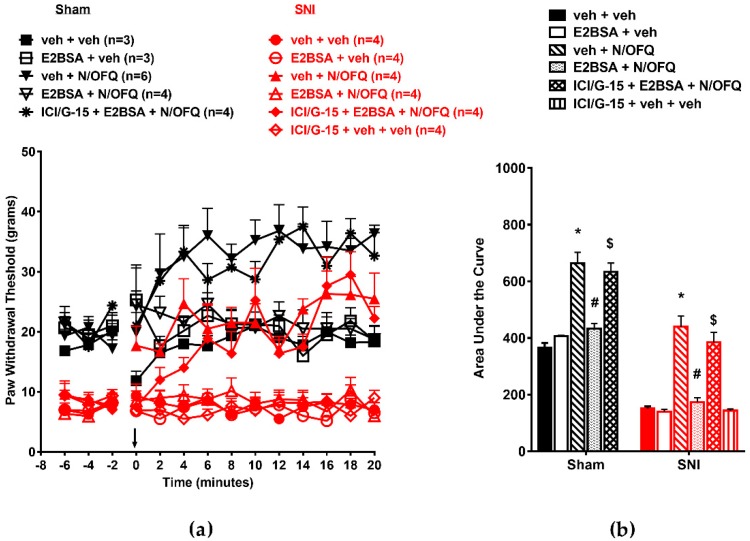
Intrathecally administered (β-estradiol 6-(*O*-carboxymethyl) oxime/bovine serum albumin (BSA)) (E_2_BSA) rapidly attenuated nociceptin/orphanin FQ (N/OFQ) peptide receptor (NOP)-mediated antihypersensitivity in ovariectomized (OVX) rats: (**a**) Spared nerve injury (SNI) significantly reduced paw withdrawal thresholds (PWTs) compared to the sham group. N/OFQ (10 nM) increased PWTs in both the sham and SNI groups. Co-administration with E_2_BSA (0.5 mM) abolished the N/OFQ-induced increase in PWTs. Pretreatment with membrane estrogen receptor (mER) antagonist (ICI 182,780 and G-15 cocktail) restored an N/OFQ-induced increase in PWTs. (**b**) the area under the curve (AUC) analysis confirmed these effects, with a significantly reduced AUC in the SNI group, N/OFQ significantly increasing it, and E_2_BSA attenuating the effect of N/OFQ in an antagonist-reversible manner. Here, * *p* < 0.05 compared to veh + veh; # *p* < 0.05 compared to veh + N/OFQ; $ *p* < 0.05 compared to E_2_BSA + N/OFQ.

First, in OVX animals, SNI led to a significant reduction in PWTs throughout the time course compared to the sham group (*F*_(130,429)_ = 2.18; *p* < 0.05), which was indicative of nerve injury-induced tactile hypersensitivity (Figure 1a). Intrathecal administration of N/OFQ significantly increased PWTs compared to the vehicle-injected group at all time points (*p* < 0.05), which was indicative of NOP-mediated antihypersensitivity. E_2_BSA co-administration with N/OFQ led to a significant reduction in PWTs compared to the N/OFQ-injected SNI group, which was indicative of a complete reversal of N/OFQ-induced antihypersensitivity. In the sham group, N/OFQ increased PWTs from baseline levels at time points 4–20 (*p* < 0.05), which was indicative of antinociception. Co-administration of E_2_BSA with N/OFQ reduced PWTs to baseline levels in both groups at all time points (*p* < 0.05). The effect of E_2_BSA in N/OFQ-treated groups was blocked by the mER antagonist cocktail ICI-182,780/G-15, while the antagonist cocktail or E_2_BSA did not have an effect when injected alone (Figure 1a). AUCs were calculated from time course plots to obtain a single measure of the overall drug response. The time course plots showed they were affected similarly (*F*_(10,43)_ = 46.51; *p* < 0.05; Figure 1b), with SNI significantly reducing the AUC, N/OFQ causing a significant increase, and E_2_BSA reversing this increase in the sham and SNI groups compared to their respective controls (*p* < 0.05).

**Figure 2:**
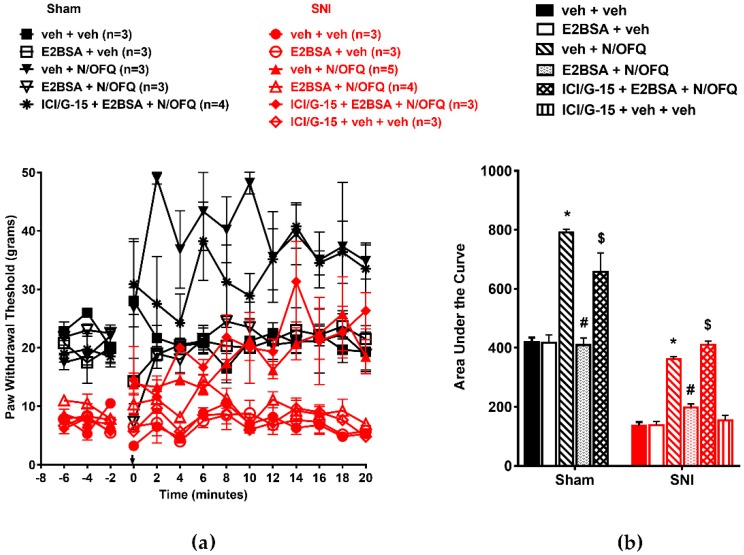
Intrathecally administered E_2_BSA rapidly attenuated NOP-mediated antihypersensitivity in male rats. (**a**) SNI significantly reduced PWTs compared to the sham group. N/OFQ (10 nM) increased PWTs in both the sham and SNI groups. Co-administration of E_2_BSA (0.5 mM) abolished the N/OFQ-induced increase in PWTs. Pretreatment with mER antagonist (ICI 182,780 and G-15 cocktail) restored an N/OFQ-induced increase in PWTs. (**b**) AUC analysis confirmed these effects, with a significantly reduced AUC in the SNI groups, N/OFQ significantly increasing it, and E_2_BSA attenuating the effect of N/OFQ in an antagonist-reversible manner. Here, * *p* < 0.05 compared to veh + veh; # *p* < 0.05 compared to veh + N/OFQ; $ *p* < 0.05 compared to E_2_BSA + N/OFQ.

In male animals, we observed similar effects of N/OFQ and E_2_BSA on PWTs as in OVX animals (Figure 2a). Intrathecal N/OFQ significantly increased PWTs in the sham group and reversed SNI-induced decreases in PWTs (*F*_(130,338)_ = 2.09; *p* < 0.05). E_2_BSA co-administration blocked the effect of N/OFQ (*p* < 0.05; Figure 2a). The AUC was affected similarly (*p* < 0.05; Figure 2b). 

These data were consistent with the interpretation that simultaneous activation of multiple mERs (ERα, ERβ, GPR30, and Gq-mER) rapidly attenuates NOP-mediated antinociception and tactile antihypersensitivity following SNI. We next investigated the selective contribution of each mER to the observed effect by using receptor-selective ligands. 

### 3.2. Selective Activation of ERα Rapidly Attenuated NOP-Mediated Tactile Antihypersensitivity

**Figure 3:**
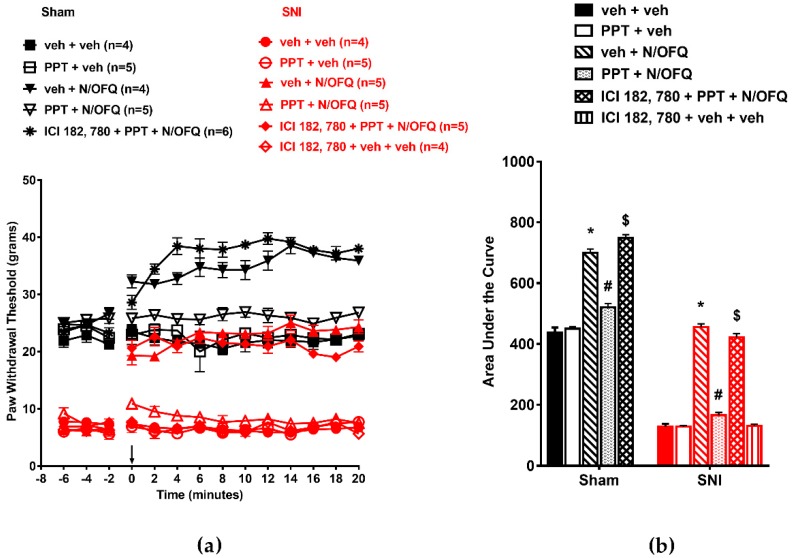
Selective activation of ERα attenuated NOP-mediated antihypersensitivity in OVX rats: (**a**) SNI significantly reduced PWTs compared to the sham group. Intrathecal administration of N/OFQ (10 nM) significantly increased paw withdrawal thresholds, whereas propylpyrazoletriol (PPT) (100 nM), the selective ERα agonist, attenuated the N/OFQ-induced increase in PWTs. Pretreatment with mER antagonist (ICI 182,780) restored an N/OFQ-induced increase in PWTs. (**b**) AUC analysis confirmed these effects, with a significantly reduced AUC in SNI groups, N/OFQ significantly increasing it, and PPT attenuating the effect of N/OFQ in an antagonist-reversible manner. Here, * *p* < 0.05 compared to veh + veh; # *p* < 0.05 compared to veh + N/OFQ; $ *p* < 0.05 compared to PPT + N/OFQ.

In OVX animals, co-administration of PPT, a selective agonist at ERα, with N/OFQ quickly attenuated N/OFQ-induced increase in PWT (Figure 3a). SNI significantly reduced PWTs as compared to the sham group (*p* < 0.05) indicating tactile hypersensitivity. Intrathecal N/OFQ led to antihypersensitivity as seen by a significant increase in PWTs which lasted the duration of nociceptive testing (*p* < 0.05). In sham animals, N/OFQ increased PWT from baseline from time point 0 to 20 (*p* < 0.05). The ER antagonist ICI-182, 780 was able to block the effect of PPT in N/OFQ treated rats (Figure 3a). Similar effects were seen in the AUCs (*F*_(10,51)_=462.77; *p* < 0.05; Figure 3b).

**Figure 4:**
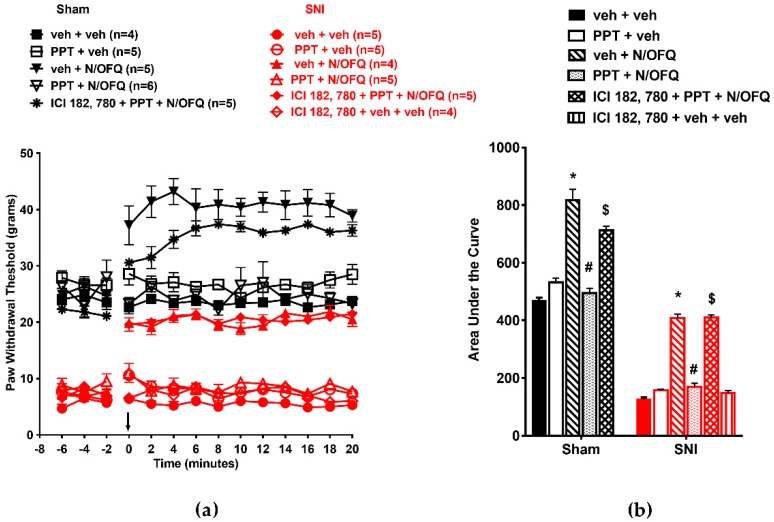
Selective activation of ERα in male rats rapidly attenuated NOP-mediated antihypersensitivity: (**a**) SNI significantly reduced PWTs compared to the sham group. N/OFQ (10 nM) increased PWTs in both the sham and SNI groups. PPT (100 nM) abolished the N/OFQ-induced increase in PWTs. Pretreatment with mER antagonist (ICI 182,780) restored an N/OFQ-induced increase in PWTs. (**b**) AUC analysis confirmed these effects, with a significantly reduced AUC in the SNI groups, N/OFQ significantly increasing it, and PPT attenuating the effect of N/OFQ in an antagonist-reversible manner. Here, * *p* < 0.05 compared to veh + veh; # *p* < 0.05 compared to veh + N/OFQ; $ *p* < 0.05 compared to PPT + N/OFQ.

In male animals, we observed similar effects of N/OFQ and PPT on PWTs as in OVX animals (Figure 4a). SNI-induced tactile hypersensitivity was attenuated by N/OFQ (*F*_(130,546)_ = 39.21; *p* < 0.05), and PPT co-administration abolished the effect of N/OFQ (*p* < 0.05; Figure 4a). The AUCs were similarly affected (Figure 4b; *F*_(10,53)_ = 209.92; *p* < 0.05). The results suggest that activation of spinal ERα alone is sufficient to disrupt NOP-mediated antinociception in sham animals and antihypersensitivity in nerve-injured OVX and male animals. 

### 3.3. Selective Activation of ERβ Rapidly Abolished the Effect of N/OFQ

**Figure 5:**
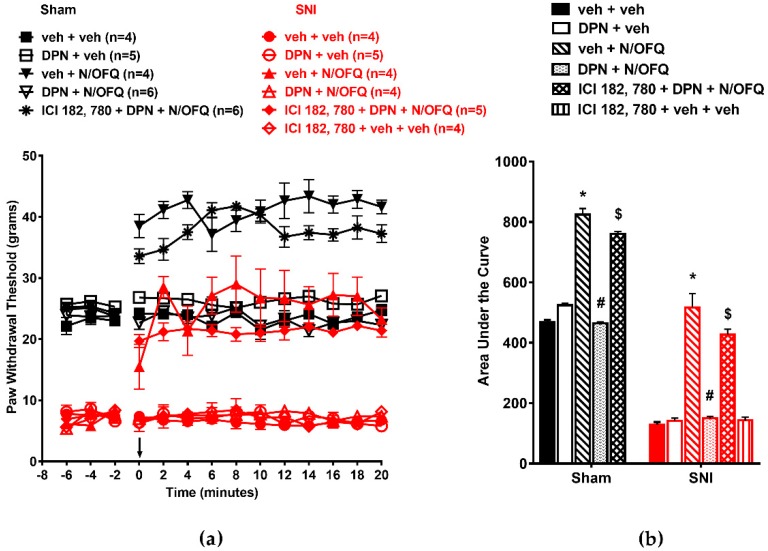
Selective activation of ERβ attenuated NOP-mediated antihypersensitivity in OVX female rats: (**a**) SNI of the sciatic nerve significantly reduced PWTs compared to the sham group. Intrathecal administration of N/OFQ (10 nM) significantly increased paw withdrawal thresholds, and diarylpropionitrile (DPN) (100 nM), the selective ERβ agonist, attenuated the N/OFQ-induced increase in PWTs. Pretreatment with mER antagonist (ICI 182,780) restored the N/OFQ-induced increase in PWTs. (**b**) AUC analysis confirmed these effects, with a significantly reduced AUC in SNI groups, N/OFQ significantly increasing it, and DPN attenuating the effect of N/OFQ in an antagonist-reversible manner. Here, * *p* < 0.05 compared to veh + veh; # *p* < 0.05 compared to veh + N/OFQ; $, *p* < 0.05 compared to DPN + N/OFQ.

Next, we explored the effect of selective ERβ activation on NOP-mediated tactile hypersensitivity. In OVX animals, SNI significantly reduced PWT compared to the sham group (*F*_(130,520)_ = 9.05; *p* < 0.05; Figure 5a). Intrathecal injection of N/OFQ significantly increased the PWT in the SNI group compared to vehicle injection (*p* < 0.05). Co-administration with DPN, the ERβ-selective agonist, led to a significant reduction in PWTs compared to N/OFQ alone (*p* < 0.05). N/OFQ injection in the sham group also significantly increased PWTs above baseline (*p* < 0.05), and co-injection of DPN blocked the effect of N/OFQ at time points 0–20 (*F*_(13,520)_ = 42.94; *p* < 0.05). This effect of DPN was reversed by ICI-182,780 (Figure 5a). The AUCs were affected in a similar manner (*F*_(10,50)_ = 243.97; *p* < 0.05; Figure 5b). 

**Figure 6:**
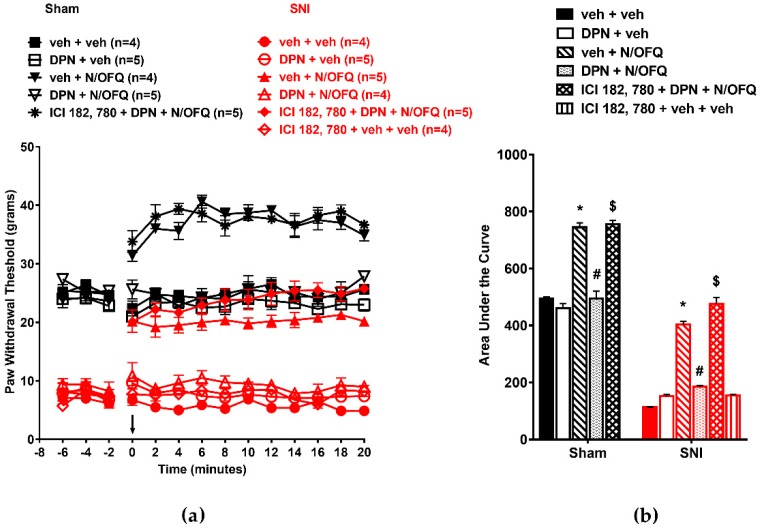
Selective activation of ERβ in male rats rapidly attenuated NOP-mediated antihypersensitivity: (**a**) SNI significantly reduced PWTs compared to the sham group. N/OFQ (10 nM) increased PWTs in both the sham and SNI groups. DPN (100 nM) abolished the N/OFQ-induced increase in PWTs. Pretreatment with mER antagonist (ICI 182,780) restored an N/OFQ-induced increase in PWTs. (**b**) AUC analysis confirmed these effects, with a significantly reduced AUC in SNI groups, N/OFQ significantly increasing it, and DPN attenuating the effect of N/OFQ in an antagonist-reversible manner. Here, * *p* < 0.05 compared to veh + veh; #, *p* < 0.05 compared to veh + N/OFQ; $, *p* < 0.05 compared to DPN + N/OFQ.

In male animals, activation of ERβ using DPN resulted in comparable effects (Figure 6a). N/OFQ reversed the SNI-induced reduction in PWTs (*F*_(130,507)_ = 6.96; *p* < 0.05), and DPN co-administration blocked the effect of N/OFQ (*p* < 0.05; Figure 6a). Similar effects were observed in the AUCs (Figure 6b; *F*_(10,48)_ = 242.87; *p* < 0.05). 

### 3.4. Selective Activation of GPR30 Rapidly Attenuated the Effect of N/OFQ

**Figure 7:**
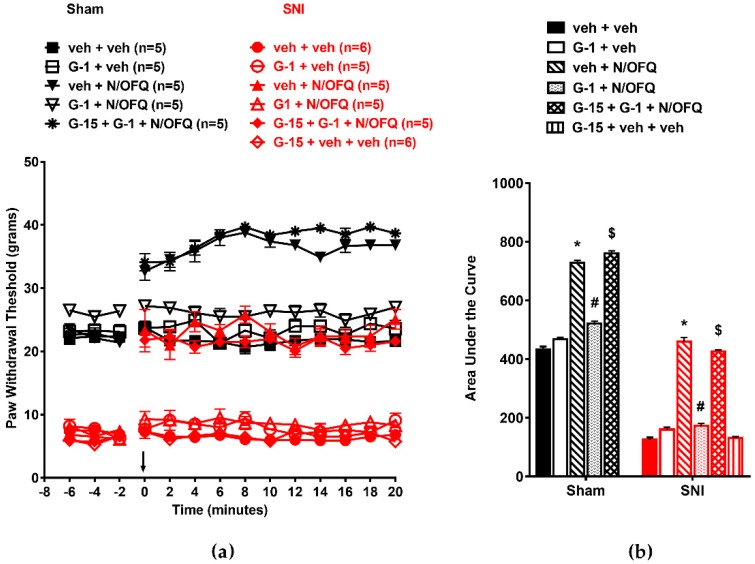
Selective activation of GPR30 attenuated NOP-mediated antihypersensitivity in OVX female rats: (**a**) SNI significantly reduced PWTs compared to the sham group. N/OFQ (10 nM) increased PWTs in both the sham and SNI groups. Co-administration of N/OFQ with G-1 (0.25 nM), the selective agonist for GPR30, abolished the N/OFQ-induced increase in PWTs. Pretreatment with GPR30 antagonist (G-15) restored an N/OFQ-induced increase in PWTs. (**b**) AUC analysis confirmed these effects, with a significantly reduced AUC in the SNI groups, N/OFQ significantly increasing it, and G-1 attenuating the effect of N/OFQ in an antagonist-reversible manner. Here, * *p* < 0.05 compared to veh + veh; #, *p* < 0.05 compared to veh + N/OFQ; $, *p* < 0.05 compared to G-1 + N/OFQ.

Next, we determined the effect of GPR30 activation on NOP-mediated antihypersensitivity. In OVX animals, SNI significantly reduced PWTs compared to the sham group (*F*_(130,598)_ = 14.88; *p* < 0.05), and N/OFQ injection increased PWTs, indicating a reversal of SNI-induced hypersensitivity. Co-administration of G-1, a selective agonist of GPR30, with N/OFQ completely blocked N/OFQ-mediated increases in PWTs (*p* < 0.05). In sham animals, N/OFQ also increased PWTs from baseline levels at time points 0–20 (*p* < 0.05), which was indicative of antinociception, and co-administration of G-1 with N/OFQ reduced PWTs to baseline levels at all time points (*p* < 0.05). Blocking GPR30 with the selective antagonist G-15 restored the effect of N/OFQ (Figure 7a). The AUCs were affected similarly; *p* < 0.05; Figure 7b).

**Figure 8:**
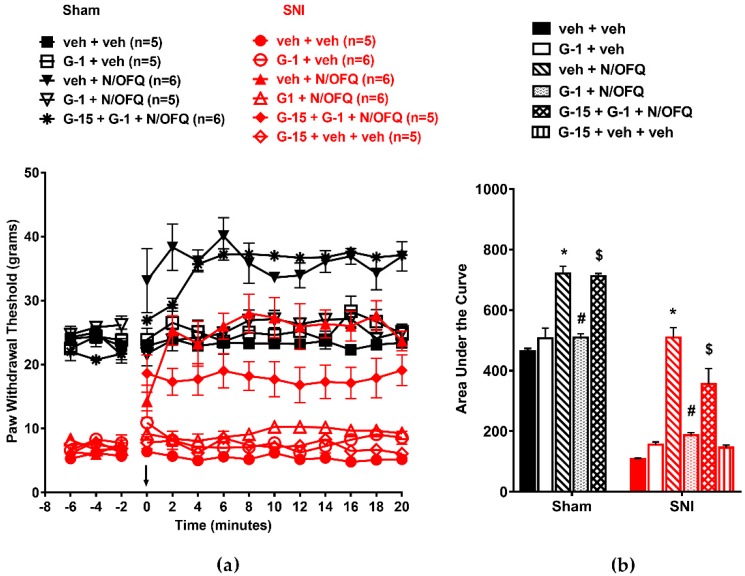
Selective activation of GPR30 attenuated NOP-mediated antihypersensitivity in male animals: (**a**) SNI significantly reduced PWTs compared to the sham group. N/OFQ (10 nM) increased PWTs in both the sham and SNI groups. G-1 (0.25 nM) abolished the N/OFQ-induced increase in PWTs. Pretreatment with GPR30 antagonist (G-15) restored an N/OFQ-induced increase in PWTs. (**b**) AUC analysis confirmed these effects, with a significantly reduced AUC in the SNI groups, N/OFQ significantly increasing it, and G-1 attenuating the effect of N/OFQ in an antagonist-reversible manner. Here, * *p* < 0.05 compared to veh + veh; #, *p* < 0.05 compared to veh + N/OFQ; $, *p* < 0.05 compared to G-1 + N/OFQ.

In male animals, intrathecally administered N/OFQ reversed SNI-induced tactile hypersensitivity (*F*_(130,637)_ = 7.10; *p* < 0.05), and G-1 co-administration blocked this effect (*p* < 0.05; Figure 8a). The AUCs were similarly affected (*F*_(10,59)_ = 99.82; *p* < 0.05; Figure 8b).

### 3.5. Selective Activation of Gq-mER Rapidly Abolished the Effects of N/OFQ

**Figure 9:**
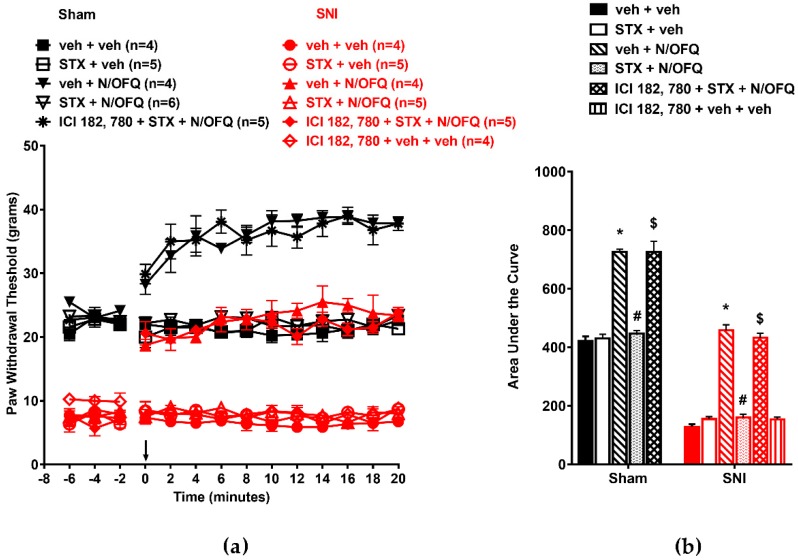
Selective activation of Gq-mER attenuated NOP-mediated antihypersensitivity in OVX rats: (**a**) SNI of the sciatic nerve significantly reduced PWTs compared to the sham group. Intrathecal administration of N/OFQ (10 nM) significantly increased PWTs, whereas STX (10 nM), the selective agonist for Gq-mER, attenuated the N/OFQ-induced increase in PWTs. Pretreatment with mER antagonist (ICI 182,780) restored an N/OFQ-induced increase in PWTs. (**b**) AUC analysis confirmed these effects, with a significantly reduced AUC in the SNI groups, N/OFQ significantly increasing it, and STX attenuating the effect of N/OFQ in an antagonist-reversible manner. Here, * *p* < 0.05 compared to veh + veh; #, *p* < 0.05 compared to veh + N/OFQ; $, *p* < 0.05 compared to STX + N/OFQ.

The role of Gq-mER activation was determined using the selective ligand STX. In OVX animals, SNI significantly reduced the PWTs compared to the sham group (*F*_(130,520)_ = 12.45; *p* < 0.05; Figure 9a). N/OFQ administered intrathecally significantly increased PWTs compared to the vehicle-treated group (*p* < 0.05). STX co-administration inhibited the N/OFQ-induced increase in PWTs in the SNI group (*p* < 0.05). In sham animals, N/OFQ increased PWTs above baseline at time points 0–20 (*F*_(13,520)_ = 51.14; *p* < 0.05), and STX co-administration blocked the N/OFQ-induced increase in PWTs (*p* < 0.05). Blocking Gq-mER with ICI-182,780 restored the effect of N/OFQ (Figure 9a). Similar effects were observed on the AUCs (*F*_(10,50)_ = 160.38; *p* < 0.05; Figure 9b). 

**Figure 10:**
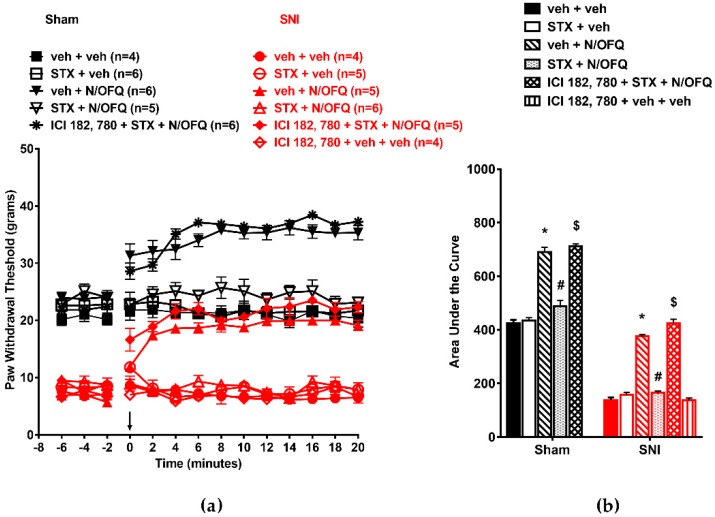
**Figure 10**. NOP-mediated antihypersensitivity was rapidly attenuated by Gq-mER activation in male rats: (**a**) SNI significantly reduced PWTs compared to the sham group. N/OFQ (10 nM) increased PWTs in both the sham and SNI groups. STX (10 nM) abolished the N/OFQ-induced increase in PWTs. Pretreatment with mER antagonist (ICI 182,780) restored an N/OFQ-induced increase in PWTs. (**b**) AUC analysis confirmed these effects, with a significantly reduced AUC in the SNI groups, N/OFQ significantly increasing it, and STX attenuating the effect of N/OFQ in an antagonist-reversible manner. Here, * *p* < 0.05 compared to veh + veh; # *p* < 0.05 compared to veh + N/OFQ; $, *p* < 0.05 compared to STX + N/OFQ. Taken together, these behavioral data suggest that simultaneous or selective activation of any spinal mER rapidly attenuated spinal NOP-mediated antinociception in the sham groups and tactile antihypersensitivity in the nerve-injured OVX female and male rats.

Similarly, in male animals, intrathecal administration of N/OFQ significantly increased PWTs in the sham and SNI groups (*F*_(130,585)_ = 11.47; *p* < 0.05). STX co-administration blocked this effect (*p* < 0.05; Figure 10a). The AUCs were comparably affected (F_(10,55)_ = 283.77; *p* < 0.05; Figure 10b). 

### 3.6. Activation of mERs Attenuated NOP-Mediated Tactile Antihypersensitivity via an ERK-, PKA-, PKC-, and Akt- Independent Mechanism

PKA, PKC, ERK I/II, and Akt play a role in central sensitization and can also be activated by estrogen [30,31,32,33,34,35] and nerve injury [36,37,38,39]. Therefore, we measured spinal levels of total and phosphorylated ERKI/II, PKA, PKC, and Akt in sham and SNI-operated OVX and male rats treated with vehicle or E_2_BSA. A densitometry analysis revealed an expected robust increase in the phosphorylation of spinal ERK I/II (*F*_(3,12)_ = 5.39; *p* < 0.05), PKA (*F*_(3,12)_ = 21.5; *p* < 0.05), PKC (*F*_(3,12)_ = 45.46; *p* < 0.05), and Akt (*F*_(3,12)_ = 18.10; *p* < 0.05) in the SNI groups compared to the sham controls. In addition, phosphorylation of PKC and Akt was higher in vehicle-treated sham (*p* < 0.05; *p* < 0.05) and SNI (*p* < 0.05; *p* < 0.05) males compared to OVX females. However, we were unable to detect any further significant increase in phosphorylation of these molecules in response to E_2_BSA administration. Data from this immunoblotting experiment are presented in a supplemental figure (Figure S1).

## 4. Discussion

This study is the first to demonstrate that (i) concomitant or selective activation of any of the four spinal mERs abolishes NOP-mediated tactile antihypersensitivity in a neuropathic pain model through a rapid mechanism; (ii) in contrast to our previous study revealing the failure of ERβ activation to attenuate NOP-mediated antinociception using an acute assay of pain, our present results suggest that ERβ activation effectively attenuates NOP-mediated tactile antihypersensitivity in a neuropathic pain model; (iii) the effect of mER activation on NOP-induced tactile antihypersensitivity is identical in both male and female sexes; and (iv) a rapid mechanism, independent of PKA, PKC, ERK I/II, or Akt activation, may underlie the effect of mER activation. 

NOP receptor activation has been pursued as a promising analgesic treatment due to the lack of several side effects that are associated with µ-opioid receptor-targeted drugs [5,6]. In preclinical studies, supraspinal administration of N/OFQ has been shown [40,41] to induce pro-nociception, whereas intrathecal administration induces antinociception [5,42]. Our findings of intrathecal N/OFQ leading to an increase in mechanical thresholds in sham animals and inducing tactile antihypersensitivity in nerve-injured rats are consistent with the antinociceptive effects of N/OFQ observed in other studies, including our own [13,18,42,43,44]. Our present results extend the previous findings of sex-related differences and estrogen-induced attenuation of NOP-mediated acute antinociception [13,18] to mER (concomitant or individual) activation-induced attenuation of NOP-mediated tactile antihypersensitivity in a rodent model of neuropathic pain. This effect was observed in both sexes upon mER activation. However, since the physiological level of estrogen in naïve males is low relative to females, it is not expected to cause significant activation of mER and hinder NOP-mediated antinociception. 

We have previously shown that spinal administration of estrogen abolishes N/OFQ-induced antinociception in acute thermal pain as well as thermal hyperalgesia models [13,18]. In addition, we reported an mER activation-induced, ERK-dependent, nongenomic pathway underlying estrogen-induced rapid attenuation of NOP-mediated antinociception [18]. This pathway was inducible by ERα, GPR30, and Gq-mER, but not by ERβ. However, our present findings reveal that all four mERs, including ERβ, effectively attenuated N/OFQ-induced tactile antihypersensitivity. This contrasting effect of ERβ under two different pain conditions cannot be explained with the current set of data. However, we believe that the sensitized state of the central nervous system (CNS) following nerve injury may facilitate mechanisms enabling ERβ to produce the observed effect.

Our results revealed that concomitant activation of all spinal mERs using E_2_BSA led to rapid attenuation of N/OFQ-induced tactile antihypersensitivity. Our previous study [18] demonstrated that ERK activation was required for the attenuation of NOP’s antinociceptive effect in an acute pain model. In the present study, SNI expectedly increased the activation of PKA, PKC, ERK I/II, and Akt: However, mER activation failed to further increase these levels. In contrast, a recent report has shown mER-induced increases in the activation of PKA, PKC, and Akt, leading to the attenuation of NOP-mediated inhibition of proopiomelanocortin (POMC) neurons in female rats [45]. We believe that in the present study, nerve injury maximally activated ERK I/II, PKA, PKC, and Akt. Hence, mER activation failed to further increase them. Secondly, the measurement of kinase activation in pain processing neurons in the spinal dorsal horn may have yielded mER-induced changes that were likely diluted and thus were not observed in the immunoblot analysis of whole lumbosacral spinal tissue in the present study. This will require further investigation.

We did observe higher activation of PKC and AKT in vehicle-treated control male animals compared to OVX animals. There has been no prior report of such differences: In fact, there was no difference in PKC activation in our previous study. Therefore, these observations remain unexplained at this time: However, sex-related differences might still exist in PKC and AKT activation, and further experiments, including intact male and female groups, will be required to address this issue. 

Finally, we report that selective activation of individual mERs was just as effective as the concomitant activation of all four mERs (ERα, ERβ, GPR30, and Gq-mER) in attenuating N/OFQ-induced tactile antihypersensitivity. It has been demonstrated that estrogen can modulate nociceptive regulatory mechanisms. The rapid actions of estrogen in various cell types are well-documented [26,46,47,48,49] and are typically attributed to membrane estrogen receptors [50,51,52]. The activation of mERs initiates a host of intracellular signaling cascades in various systems [26,53,54], but those involved in mediating the rapid modulation of spinal pain and analgesia remain largely unknown. ERα and ERβ mRNA have been colocalized with NOP in the spinal dorsal horn, providing the cellular basis for their interaction [55]. GPR30 has been established as a main mediator of rapid estrogenic effects [56,57,58]. GPR30 is mainly a membrane-dwelling receptor [59,60] and has been localized in the spinal dorsal horn [46], which suggests a likely interaction with NOP and a possible mechanism for GPR30 activation-induced attenuation of N/OFQ’s effect. Gq-mER is also a membrane-bound receptor [27]. Although its distribution in the spinal dorsal horn has not been studied yet due to a lack of selective antibodies, a recent study reported that NOP-mediated inhibition of proopiomelanocortin (POMC) neurons in the hypothalamus was attenuated by STX [45]. Our results are consistent with this finding as well as with a similar effect of STX reported in our previous study [18]. 

Interestingly, the effect of mER activation in male animals was similar to that in females. These findings are consistent with our previous findings [18]. In addition, mERs are also present in the spinal dorsal horn of male rats [61] and are therefore expected to be activated by intrathecally injected agonists to effectively attenuate NOP-induced tactile antihypersensitivity. Physiologically, however, the low level of circulating estrogen in males is not expected to activate mERs to produce a significant effect on NOP-induced antihypersensitivity.

## 5. Conclusions

Overall, our findings highlight mER activation-induced, rapid attenuation of NOP-mediated tactile antihypersensitivity in a neuropathic model of pain. A blockade of mERs may present an effective strategy to improve GPCR-mediated analgesia in women.

## Supplementary Materials

The following are available online at https://www.mdpi.com/2076-3425/9/6/147/s1, Figure S1: Activation of mERs attenuated NOP-mediated tactile antihypersensitivity via an ERK-, PKA-, PKC-, and Akt-independent mechanism.
