# Supplementary material for: Activation of Membrane Estrogen Receptors Attenuates NOP-Mediated Tactile Antihypersensitivity in a Rodent Model of Neuropathic Pain
Source: Brain Sci. 2019 Jun 21;9(6):147. doi: 10.3390/brainsci9060147 (PMC6628583; doi:10.3390/brainsci9060147)
Supplement: Supplementary file 1 [file brainsci-09-00147-s001.pdf]

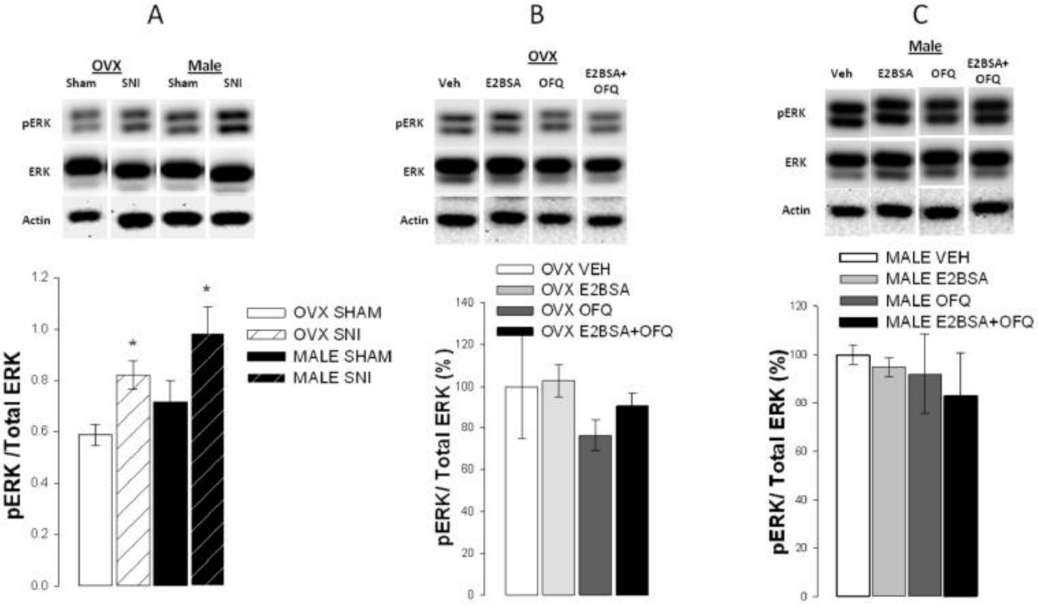
A
OVX
Male
Sham
SNI
Sham
SNI
pERK
ERK
Actin
pERK /Total ERK
OVX SHAM
OVX SNI
MALE SHAM
MALE SNI
B
OVX
Veh
E2BSA
OFQ
E2BSA+ OFQ
pERK
ERK
Actin
OVX VEH
OVX E2BSA
OVX OFQ
OVX E2BSA+OFQ
pERK/ Total ERK (%)
C
Male
Veh
E2BSA
OFQ
E2BSA+ OFQ
pERK
ERK
Actin
MALE VEH
MALE E2BSA
MALE OFQ
MALE E2BSA+OFQ
pERK/ Total ERK (%)

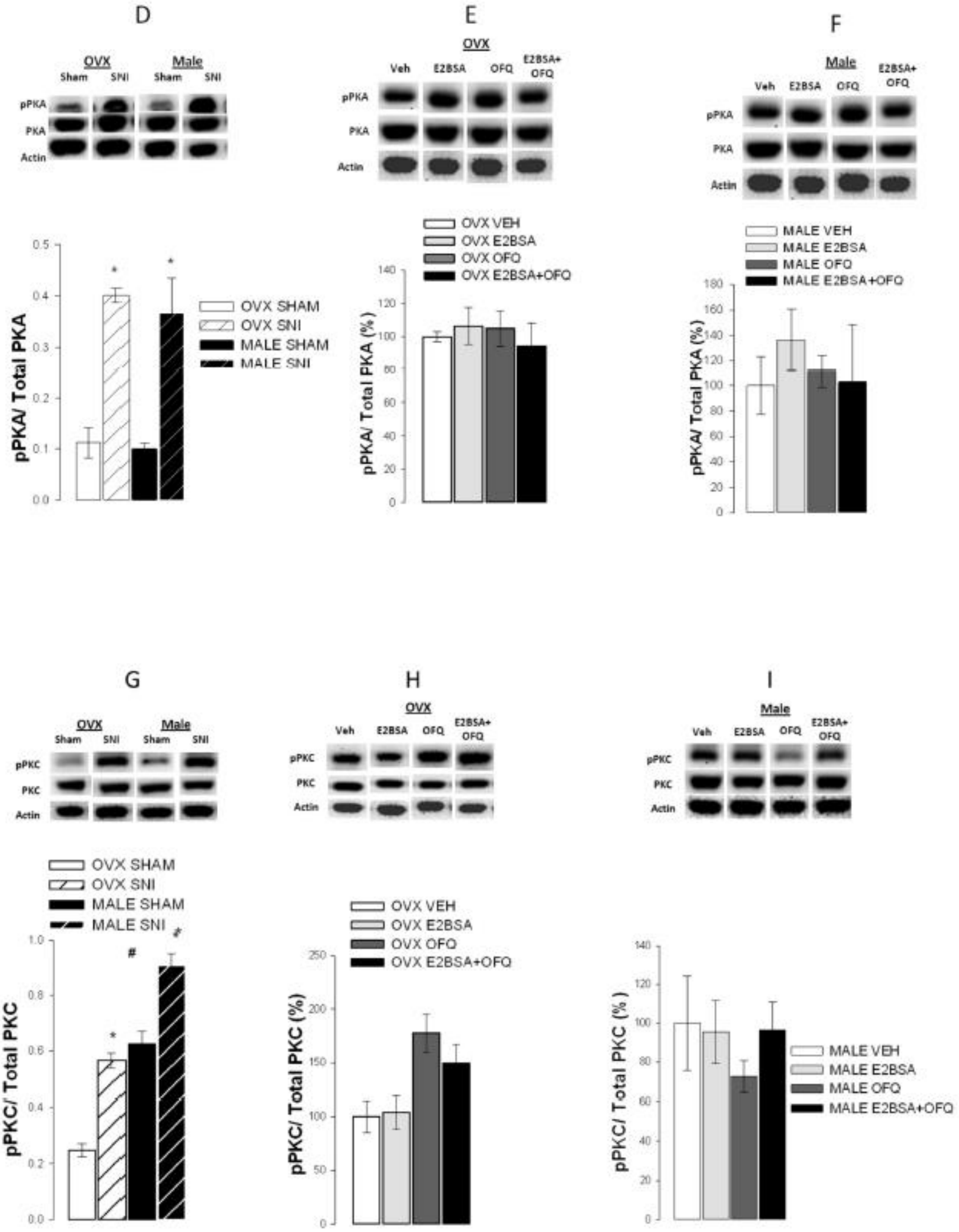
D
OVX
Male
Sham
SNI
Sham
SNI
pPKA
PKA
Actin
OVX SHAM
OVX SNI
MALE SHAM
MALE SNI
pPKA/ Total PKA
E
OVX
Veh
E2BSA
OFQ
E2BSA+ OFQ
pPKA
PKA
Actin
OVX VEH
OVX E2BSA
OVX OFQ
OVX E2BSA+OFQ
pPKA/ Total PKA (%)
F
Male
Veh
E2BSA
OFQ
E2BSA+ OFQ
pPKA
PKA
Actin
MALE VEH
MALE E2BSA
MALE OFQ
MALE E2BSA+OFQ
pPKA/ Total PKA (%)
G
OVX
Male
Sham
SNI
Sham
SNI
pPKC
PKC
Actin
OVX SHAM
OVX SNI
MALE SHAM
MALE SNI
pPKC/ Total PKC
H
OVX
Veh
E2BSA
OFQ
E2BSA+ OFQ
pPKC
PKC
Actin
OVX VEH
OVX E2BSA
OVX OFQ
OVX E2BSA+OFQ
pPKC/ Total PKC (%)
I
Male
Veh
E2BSA
OFQ
E2BSA+ OFQ
pPKC
PKC
Actin
MALE VEH
MALE E2BSA
MALE OFQ
MALE E2BSA+OFQ
pPKC/ Total PKC (%)

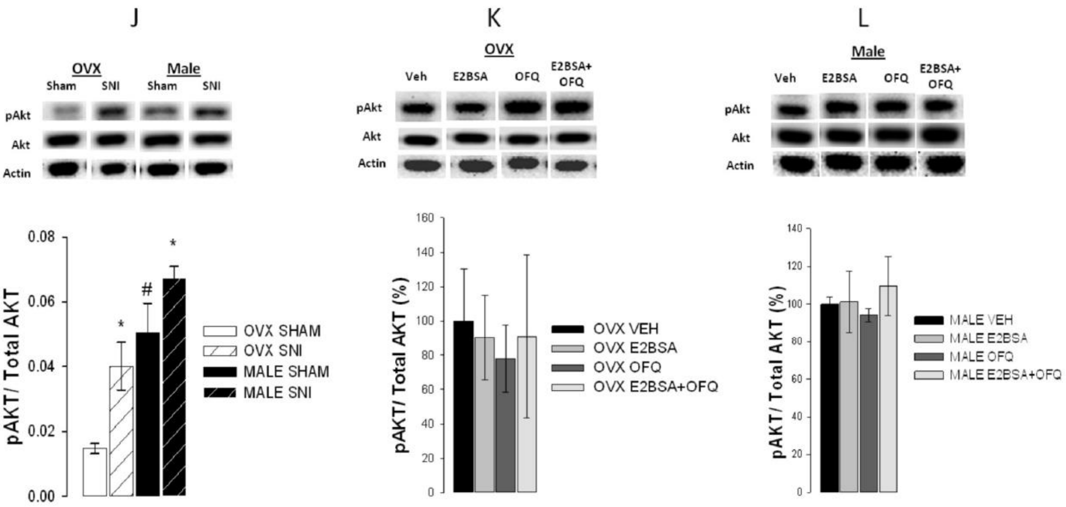


**Figure 1.** E2BSA abolishes NOP-mediated anti-hypersensitivity via an ERK, PKA, PKC, and Akt-independent mechanism. SNI induced significant increases in spinal phosphorylation of ERK (A); PKA (D); PKC (G): and Akt (J) in OVX and male rats. However, mER activation did not significantly change spinal phosphorylation of ERK (B, C); PKA (E, F); PKC (H, I); or Akt (K, L) in OVX or male rats, respectively. *$p<0.01$ compared to sham. #$p<0.01$ compared to OVX.
